# Clinical and imaging profiles of pulmonary embolism: a single-institution experience

**DOI:** 10.1186/s12245-020-00303-y

**Published:** 2020-08-31

**Authors:** Omran Al Dandan, Ali Hassan, Hossain AbuAlola, Alaa Alzaki, Abrar Alwaheed, Mohannad Alalwan, Malak Al Shammari, Nouf AlShamlan, Hind S. Alsaif

**Affiliations:** 1 Department of Radiology, King Fahd Hospital of the University, Imam Abdulrahman Bin Faisal University, Al-Khobar, Saudi Arabia; 2 Department of Internal Medicine, King Fahd Hospital of the University, Imam Abdulrahman Bin Faisal University, Al-Khobar, Saudi Arabia; 3 Department of Family and Community Medicine, College of Medicine, Imam Abdulrahman Bin Faisal University, Dammam, Saudi Arabia

**Keywords:** Computed tomography angiography, Pulmonary embolism, Sickle cell disease, Obesity

## Abstract

**Background:**

Pulmonary embolism (PE) is a common life-threatening condition with non-specific clinical presentations. The diagnosis of PE depends highly on imaging studies, which may also provide prognostic information. This study aimed to describe the clinical and imaging profiles of patients with PE, emphasizing the differences between central and peripheral PE.

**Methods:**

After ethics review board approval, this retrospective observational study examined the non-negative results in adult patients who underwent computed tomography pulmonary angiography (CT-PA) at our hospital between May 2016 and December 2019. Demographic and clinical information and imaging findings were collected from the electronic medical records.

**Results:**

The study included 85 cases that were identified after re-interpreting the 103 non-negative CT-PA scans. Six cases were excluded for incomplete data and 12 cases were false-positive. Central PE was found in 63.5% of the cases. Obesity was the most common risk factor seen in 37.6% of the cases. Furthermore, 9.4% of the patients had sickle cell disease, which tended to be associated with peripheral PE. There was no difference between the peripheral and central PE in most clinical and imaging parameters evaluated (*P* > 0.05). However, patients with isolated subsegmental PE were more likely to develop hemoptysis (*P* = 0.04).

**Conclusion:**

This study suggests that patients with obesity and sickle cell disease constitute an important proportion of all PE cases. Furthermore, the clinical and imaging profiles in patients with peripheral PE are similar to those in patients with central PE. Future research should focus on the clinical value of peripheral PE in patients with sickle cell disease.

## Background

Pulmonary embolism (PE) is a form of thromboembolic disease that is potentially life-threatening. The global burden of PE has not been estimated; however, it accounts for 100,000 deaths in the USA annually [1]. Despite being a common condition, PE can pose a diagnostic challenge because of its variable presentations. Delaying or missing the diagnosis might result in significant morbidity and mortality [1].

Although the clinical features and risk factors for PE have been widely studied, a detailed evaluation of the geographic locations of these studies reveals that most were conducted in Europe, North America, and the Far East. Little is known about the characteristics of patients with PE in the Middle East, particularly in Saudi Arabia. It would be of particular interest to explore the risk profile for PE in this uncharted area, considering the relatively higher prevalence of certain hematological disorders that may be associated with a thrombotic tendency [2, 3].

The definitive diagnosis of PE depends heavily on imaging studies, with computed tomography pulmonary angiography (CT-PA) being the imaging modality of choice. Additionally, because of its ability to establish a diagnosis of PE by demonstrating filling defects in the pulmonary vasculature, CT-PA may allow the assessment of other parameters, such as cardiovascular and pleuroparenchymal features, that are prognostically relevant [4–6].

This study sought to describe the clinical and imaging profiles of patients with PE who presented at an academic institution in the Eastern Province of Saudi Arabia. We also aimed to investigate whether there is any difference between the profiles of central and peripheral PE. The findings of this study will broaden our current knowledge of PE in Saudi Arabia.

## Methods

### Study design and setting

After ethics review board approval, we conducted a retrospective study, wherein the clinical and imaging profiles of PE were investigated. Particular emphasis was placed on the differences between central and peripheral PE. The study was conducted at the King Fahd Hospital of the Imam Abdulrahman Bin Faisal University.

### Study population

The Radiology Information System was searched to identify all the requests for CT-PA scans between May 2016 and December 2019 for both hospitalized patients and those in the Emergency Department (ED). Eligible participants were adult patients who underwent CT-PA to rule out PE. Exclusion criteria were as follows: (1) age < 18 years; (2) pregnancy; (3) purpose of scan was not to rule out PE; (4) chronic venous thromboembolism. Where more than one CT-PA scan had been carried out on the same patient within the same clinical encounter, only the most recent scan was included, as other scans were assumed to be of suboptimal image quality.

### Data collection

A structured data collection form was used to collect data from the patient electronic medical records. Data included information related to demographics, as well as clinical and imaging profiles of PE.

#### Background demographic information

Details of age, sex, comorbidities, risk factors, and clinical setting (ED vs. hospitalized patient) were collected. The Charlson Comorbidity Index (CCI) was used to assess comorbidities. The CCI score can range from 0 to 37 and includes 17 different components [7]. The assessed risk factors included immobilization, recent surgery or trauma, presence of a previous venous thromboembolic disease, smoking status, obesity, sickle cell disease, use of a central venous catheter, exogenous estrogen use, and a previously existing malignancy.

#### Clinical profile of pulmonary embolism

Data on the clinical symptoms and signs at presentation, vital signs, clinical severity, management, and outcomes were recorded. The clinical severity of PE was assessed using the Pulmonary Embolism Severity Index (PESI) [8], which is the most validated prognostic tool for PE [9].

#### Imaging profile of pulmonary embolism

Data on the chest radiographs and CT-PA scans were obtained. The images were assessed for the presence of filling defects in the pulmonary vasculature and for pleuroparenchymal abnormalities.

### Definitions

Hypoxia was defined as an oxygen saturation < 95% on room air, tachycardia was defined as a heart rate > 100 bpm, and hypotension was defined as blood pressure < 90/60 mmHg. Obesity was defined as a body mass index ≥ 30 kg/m^2^, and immobilization was defined as bed rest for more than 3 days. Recent surgery was defined as any surgery under general or regional anesthesia in the 4 weeks prior to the PE diagnosis. Central PE was defined as occurring in the pulmonary trunk, main pulmonary arteries, or lobar pulmonary arteries; peripheral PE was defined as occurring exclusively in the segmental or subsegmental pulmonary arteries. Low-risk PE was defined as corresponding to PESI classes I–II, while high-risk PE was defined as corresponding to PESI classes III–V.

### Imaging interpretation

All images were independently interpreted by two consultant radiologists, one of whom had fellowship training in chest imaging. Both radiologists were blinded to all patient information, and any discrepancies in interpretation were resolved by consensus.

### Statistical analysis

Data were compiled using the QuestionPro platform (Seattle, WA, USA) and analyzed using IBM SPSS for Windows, version 25 (IBM Corp., Armonk, NY, USA). Categorical variables are presented as percentages and frequency distributions. Continuous variables are presented as median and interquartile range (IQR). The Kolmogorov–Smirnov and Shapiro–Wilk tests were used to determine whether the data had a normal distribution. Continuous data were compared using the independent *t* test or Mann–Whitney *U* test, as appropriate. Categorical variables were compared using the chi-squared test or Fisher’s exact test, as appropriate. The significance level was set to α = 0.05. Due to the small sample size, multivariable logistic regression was not conducted.

## Results

### Patient characteristics

**Fig. 1:**
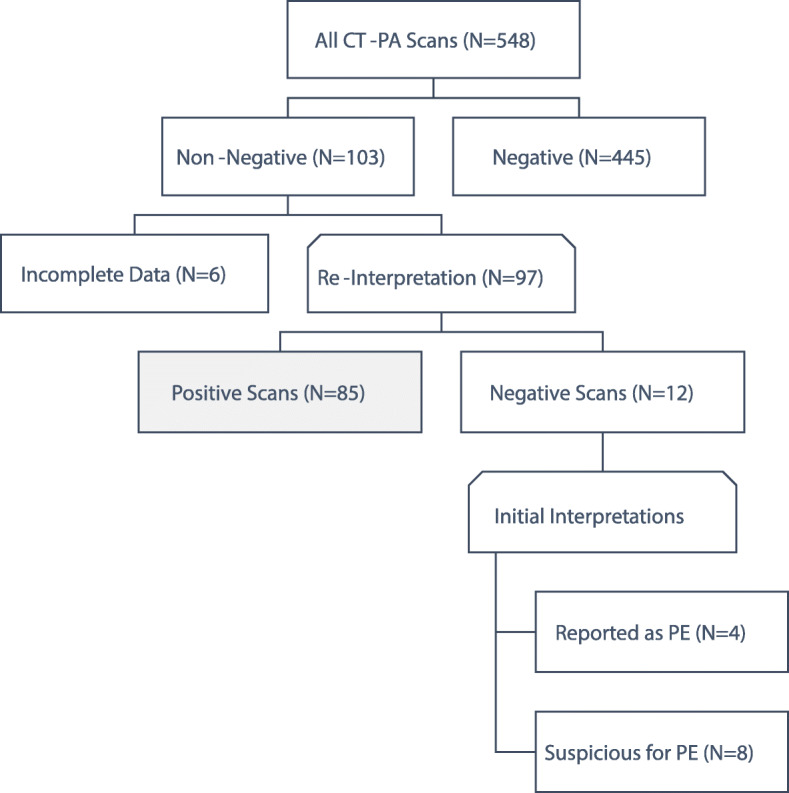
Flow chart of the study. N: number of cases; CT-PA: computed tomography pulmonary angiography; PE: pulmonary embolism

The study involved 103 CT-PA scans with PE diagnosis according to the radiology reports. Six CT-PA scans were excluded because of incomplete data. After reinterpreting the images, 12 CT-PA scans were found to not show PE. Of these 12 CT-PA scans, 4 were initially reported to show PE, and the remaining 8 were suspicious for PE. Eighty-five CT-PA scans were, therefore, deemed valid for further analysis (Fig. 1).

There were 40 men (47.1%) and 45 women (52.9%) with a median age of 42.0 years (IQR 32.5–58.5 years). Fifty-five (64.7%) presented to the ED, while 30 (35.3%) were hospitalized patients. The CCI score was 0 in 40 (47.1%), 1–2 in 21 (24.7%), and ≥ 3 in 24 (28.2%) cases.

### Localization of the pulmonary embolism

**Fig. 2:**
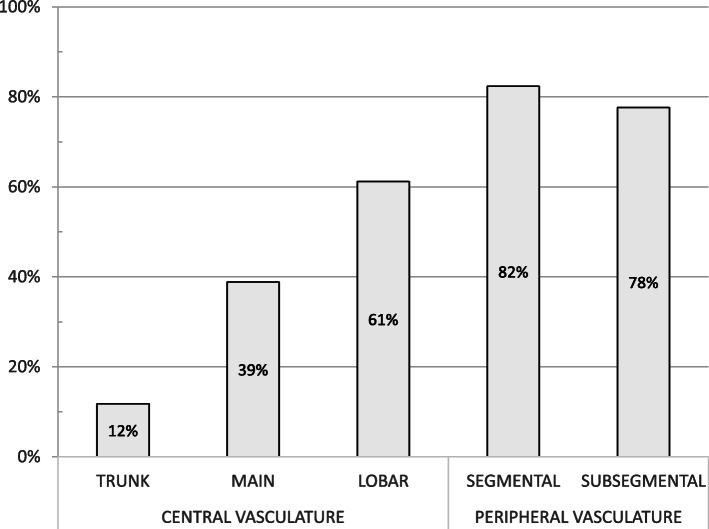
Vascular involvement of pulmonary embolism

Fifty-four PE cases (63.5%) were deemed central, and 31 (36.5%) were peripheral. Involvement of the pulmonary trunk, main pulmonary arteries, and lobar arteries was seen in 10 (11.8%), 33 (38.8%), and 52 (61.2%) cases, respectively. The segmental and subsegmental arteries were involved in 70 (82.4%) and 66 (77.6%) cases, respectively (Fig. 2), with only 8 cases having exclusive involvement of subsegmental arteries. The background characteristics of the patients with central and peripheral PE are summarized in Table 1.

**Table 1:** Background characteristics of patients with pulmonary embolism

Characteristics		Peripheral PE		Central PE		Overall
Age [median years (IQR)]		37.0 (31.0–59.0)		44.0 (32.3–59.3)		42.0 (32.5–58.5)
Gender	Male	17	(42.5%)	23	(57.5%)	40
	Female	14	(31.1%)	54	(68.9%)	45
Setting	Emergency department	17	(30.9%)	38	(69.1%)	55
	Inpatient department	14	(46.7%)	16	(53.3%)	30
Body mass index (kg/m^2^) [median (IQR)]		25.7 (20.4–30.7)		29.1 (25.0–33.8)		28.0 (22.8–32.9)
Comorbidities	Diabetes mellitus	9	(42.9%)	12	(57.1%)	21
	Myocardial infarction	2	(66.7%)	1	(33.3%)	3
	Cerebral vascular accident	3	(37.5%)	5	(62.5%)	8
	Congestive heart failure	1	(33.3%)	2	(66.7%)	3
	Chronic kidney disease	2	(66.7%)	1	(33.3%)	3
	Liver disease	2	(66.7%)	1	(33.3%)	3
	Chronic obstructive pulmonary disease	1	(50.0%)	1	(50.0%)	2
	Connective tissue disease	1	(50.0%)	1	(50.0%)	2
	Peripheral vascular disease	0	(0.0%)	1	(100.0%)	1
	Solid tumors	2	(25.0%)	6	(75.0%)	8
	Lymphoma	0	(0.0%)	2	(100.0%)	2
	Leukemia	0	(0.0%)	1	(100.0%)	1

*PE* pulmonary embolism, *IQR* interquartile range

No significant differences between central and peripheral pulmonary embolisms based on any of the included variables (*P* > 0.05)

### Severity of the pulmonary embolism

The median PESI score was 55 (IQR 37–73). The number of cases in PESI risk classes I, II, III, IV, and V were 50 (58.5%), 21 (24.7%), 5 (5.9%), 2 (2.4%), and 7 (8.2%), respectively. Seventy-one cases (83.5%) were thus in the low-risk group, and 14 cases (16.5%) were in the high-risk group. There was no difference in the severity between central and peripheral PE (*χ*^*2*^ = 0.004, *P* = 0.949).

### Risk profile for pulmonary embolism

**Fig. 3:**
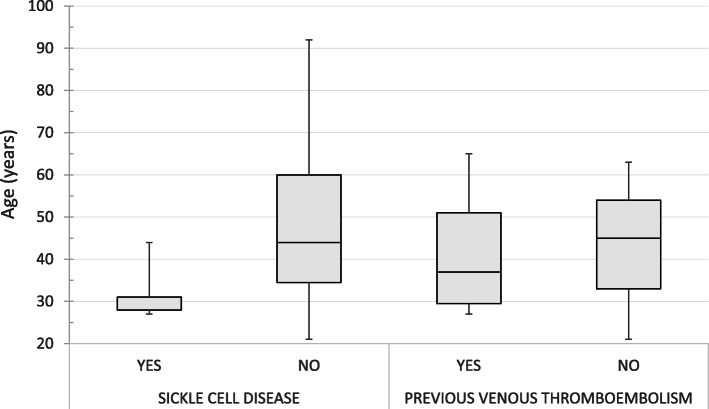
Box plots depicting the distribution of ages based on sickle cell disease and previous thromboembolism.

The majority of patients (83.5%) had at least one identified risk factor for PE, with the most common risk factor being obesity, which was present in 32 cases (37.6%). Overall, 20% of the patients had undergone a recent surgery within the preceding 4 weeks, 14 cases (16.5%) had a history of previous venous thromboembolic disease. Furthermore, 11 patients (12.9%) had malignancies, including leukemia (1.2%), lymphoma (2.4%), and solid tumors (9.4%). Sickle cell disease was a risk factor in eight cases (9.4%), of which only one was female. Both thrombophilia and the use of a central venous catheter were identified as risk factors in one unique case (1.2%). Notably, none of these risk factors was associated with the type of PE the patient developed, except sickle cell disease, in which 75% of the patients had peripheral PE (*P* = 0.047). Patients with a history of venous thromboembolism (*P* = 0.044) and sickle cell disease (*P* = 0.002) were younger than the other patients (Fig. 3).

### Clinical presentation

The most frequent presenting symptom was dyspnea, which was seen in 67 (78.8%) of the cases. Chest pain and cough were seen in 31 (36.5%) and 10 (11.2%) cases, respectively. Thirteen patients (15.3%) experienced unilateral leg swelling or pain. Hemoptysis was the least common symptom, seen in only four cases (4.7%), with only three patients (3.5%) being asymptomatic.

Tachycardia and hypoxia were seen in 43 (50.6%) and 28 (32.9%) of the cases, respectively. There were six (7.1%) patients that presented with an altered level of consciousness. Only five (5.9%) patients had hypotension. None of the presenting clinical symptoms or signs showed a significant association with the type of PE (Table 2). However, hemoptysis was more likely to occur in isolated subsegmental PE (*P* = 0.043).

**Table 2:** Clinical presentation of patients with pulmonary embolism

Characteristics		Peripheral PE		Central PE		Overall
		*N*	(%)	*N*	(%)	
Symptoms	Asymptomatic	2	(66.7)	1	(33.3)	3
	Dyspnea	25	(37.3)	42	(62.7)	67
	Chest pain	9	(29.0)	22	(71.0)	31
	Cough	6	(60.0)	4	(40.0)	10
	Hemoptysis	2	(50.0)	2	(50.0)	4
	Leg pain or swelling (unilateral)	3	(23.1)	10	(76.9)	13
	Syncope	1	(33.3)	2	(66.7)	3
Signs	Tachycardia (heart rate > 100 beats/min)	16	(37.2)	27	(62.8)	43
	Hypoxia (oxygen saturation < 95% on room air)	12	(42.9)	16	(57.1)	28
	Hypotension (blood pressure < 90/60 mmHg)	3	(60.0)	2	(40.0)	5
	Altered consciousness	2	(33.3)	4	(66.7)	6

*N* number of cases, *PE* pulmonary embolism

No significant differences between central and peripheral pulmonary embolisms based on any of the included variables (*P* > 0.05)

### Chest radiography findings

The imaging findings are summarized in Table 3. A chest radiograph was performed in 83 (97.6%) patients, and the results of which were normal in 24 (28.2%) cases. The most commonly observed abnormality was a parenchymal infiltrate, observed in 32 (37.6%) cases. Pleural effusion, cardiomegaly, and atelectasis were seen in 24 (28.2%), 18 (21.2%), and 11 (12.9%) patients, respectively. The Westermark sign was observed in only two (2.4%) cases, and the Hampton sign was not seen in any of the cases.

**Table 3:** Imaging findings of patients with pulmonary embolism

Characteristics		Peripheral PE		Central PE		Overall
		*N*	(%)	*N*	(%)	
Chest X-ray findings	Normal chest radiographic findings	5	(20.0)	19	(80.0)	24
	Parenchymal infiltrates	14	(40.0)	18	(60.0)	32
	Atelectasis	6	(50.0)	6	(50.0)	12
	Pleural effusion	11	(50.0)	13	(50.0)	24
	Cardiomegaly	9	(50.0)	9	(50.0)	18
	Pulmonary edema	3	(100.0)	0	(0.0)	3
	Venous congestion	0	(0.0)	1	(100.0)	1
	Westermark sign	0	(0.0)	2	(100.0)	2
CT-PA pleuroparenchymal findings	Normal pleuroparenchymal findings	4	(20.0)	17	(80.0)	21
	Wedge-shaped peripheral opacity	12	(30.0)	23	(70.0)	35
	Pleural effusion	11	(50.0)	12	(50.0)	23
	Enhancing atelectasis	2	(50.0)	2	(50.0)	4
	Non-enhancing atelectasis	1	(50.0)	1	(50.0)	2
	Bubbly consolidation	1	(50.0)	1	(50.0)	2

*N* number of cases, *CT-PA* computed tomography pulmonary angiography, *PE* pulmonary embolism

No significant differences between central and peripheral pulmonary embolisms based on any of the included variables (*P* > 0.05)

### Pleuroparenchymal CT-PA findings

**Fig. 4:**
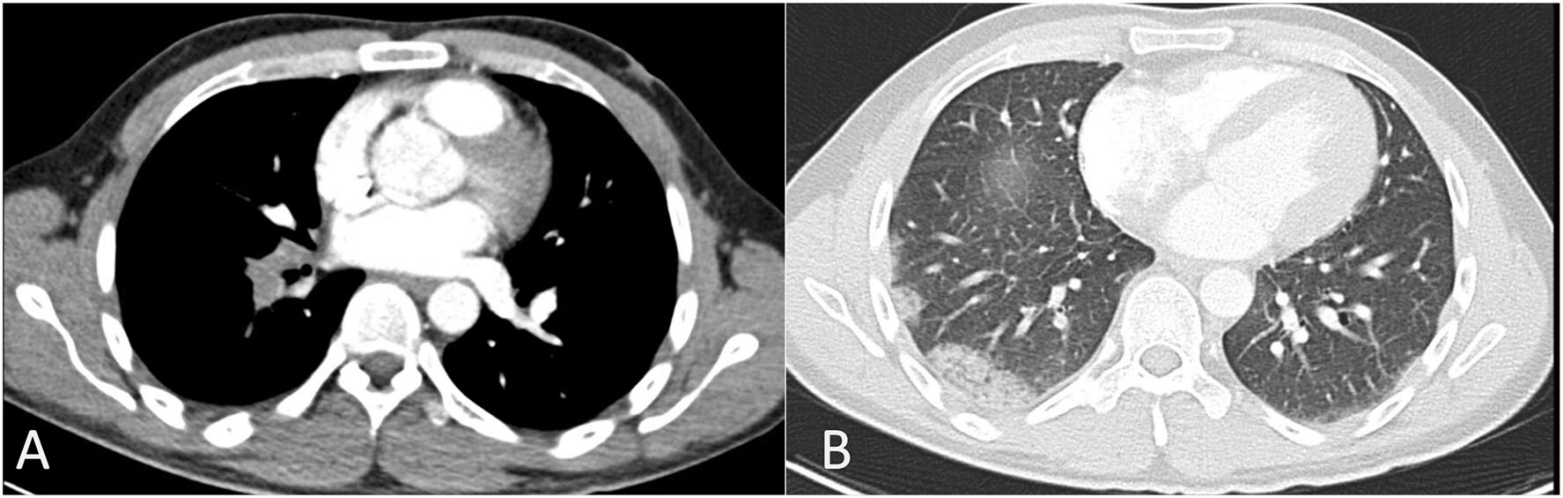
Axial computed tomography pulmonary angiography images in the pulmonary thromboembolism-specific (**a**) and lung (**b**) windows from a 32-year-old man with a history of smoking and previous venous thromboembolism presenting with chest pain and dyspnea, demonstrating a filling defect within the right pulmonary artery (**a**) and peripheral bubbly consolidation in the right lower lobe representing an infarct

**Fig. 5:**
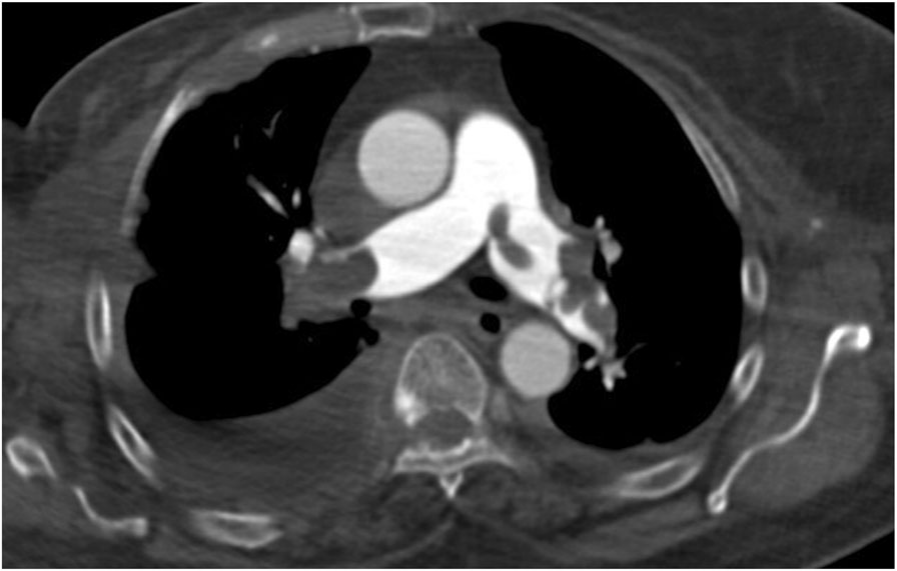
Axial computed tomography pulmonary angiography images in the pulmonary thromboembolism-specific window, from a 68-year-old woman with a history of metastatic breast cancer presenting with dyspnea and altered level of consciousness, demonstrating a filling defect within the pulmonary trunk and both main pulmonary arteries and bilateral pleural effusion that is more prominent in the right hemithorax

**Fig. 6:**
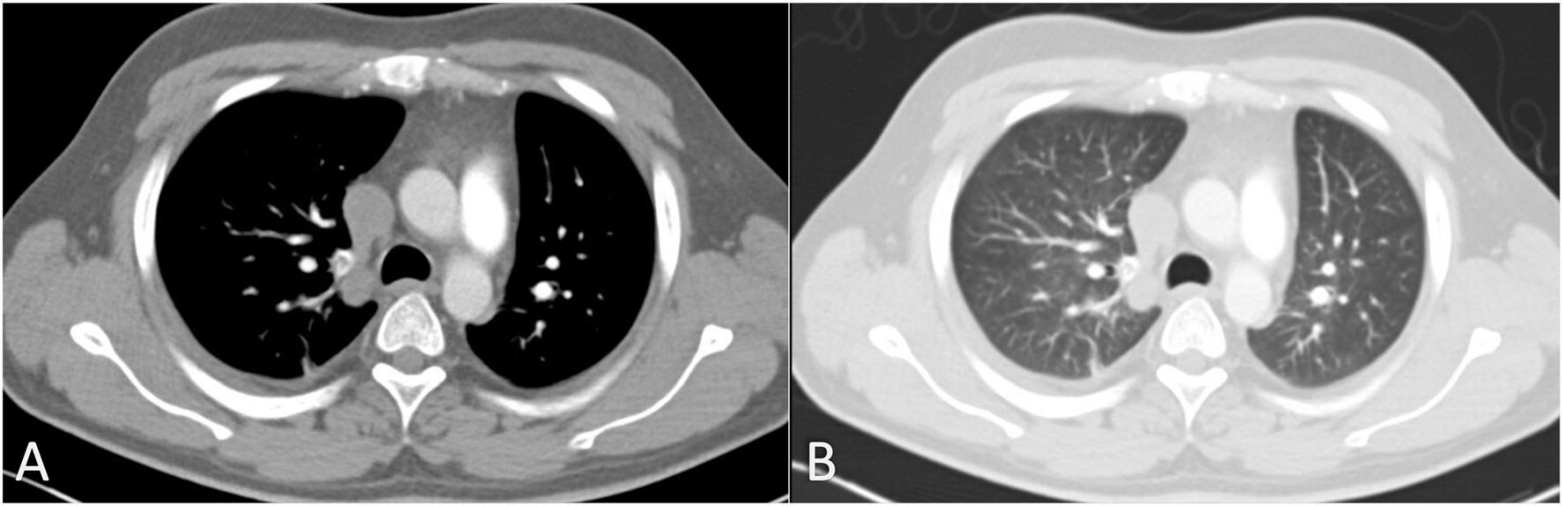
Axial computed tomography pulmonary angiography images in the pulmonary thromboembolism-specific (**a**) and lung (**b**) windows, from a 31-year-old man with sickle cell disease presenting with dyspnea, demonstrating a filling defect within the truncus anterior (**a**) and peripheral parenchymal atelectasis (**b**)

CT-PA scans showed pleuroparenchymal abnormalities in 64 (75%) cases (Figs. 4, 5 and 6). The most common pleuroparenchymal finding was that of a wedge-shaped peripheral opacity, which was seen in 35 (41.2%) cases, while pleural effusion was observed in 23 (27.1%) cases. Enhancing and non-enhancing atelectasis were seen in 4 (4.7%) and 2 (2.4%) cases, respectively, with bubbly consolidation seen in 2 (2.4%) cases. Moreover, there were no differences in the pleuroparenchymal findings between the central and peripheral PE (*P* > 0.05).

### Management options

Of the 85 cases, only 7 (8.2%) did not receive anticoagulant therapy to manage the PE. The reasons for not treating these patients with anticoagulant therapy were unclear in all except one patient, who had a history of hemorrhagic stroke as a complication of previous anticoagulant therapy, and an inferior vena cava filter put in place. The anticoagulants used were enoxaparin 32 (41.0%), rivaroxaban in 20 (25.6%), warfarin in 18 (23.1%), unfractionated heparin in 6 (7.7%), and dabigatran in 2 (2.6%) cases. The duration of treatment varied, including 1 month in 5 (5.9%), 3 months in 10 (10.6%), 6 months in 29 (34.1%), 9 months in 2 (2.4%), 12 months in 8 (9.4%), and lifelong in 11 (12.9%). Systemic thrombolysis was carried out in only 10 cases (11.7%), and none of the patients underwent surgical or mechanical thrombectomy.

### Survival and complications

The survival rate was 83.5%, as 14 (16.5%) patients had died within 30 days of the PE event. Recurrence of thromboembolic disease within 3 months was observed in a single patient with recurrent PE. Three patients (3.5%) developed bleeding events as complications due to treatment, including epistaxis, menorrhagia, and hemorrhagic stroke.

## Discussion

The study investigated the differences in the clinical and radiological profiles between patients with central and peripheral PE. The study did not find any significant differences in these patients; however, the study does demonstrate some unique features in the patients’ clinical profiles.

### Clinical profile

Our results show that half of the PE patients in this study were younger than 42 years old, an age that is notably lower than that described in the literature [10]. In the USA, a population-based study showed that the median age of patients with PE in 2012 was 65 years [10, 11]. The incidence of thromboembolism increases with age. For example, an earlier study showed that the incidence among individuals aged ≥ 70 years was three times higher than among individuals aged 45–69 years, which was, again, three times higher than the incidence rate among individuals aged 20–44 years [11]. The younger age of incidence in our study could be attributed to a high prevalence of risk factors, as more than 80% of the patients had at least one risk factor for PE. The high prevalence of risk factors in this population stresses the importance of careful history taking in patients suspected with PE.

Obesity was by far the most common risk factor for PE, seen in one-third of our patients. Obesity is a global epidemic, and Saudi Arabia is no exception [12]. It was suggested that the thrombogenic effect of obesity is partly mediated by factor VIII-related activated protein C resistance [13].

Recent surgery was found to be a high-risk factor in our study; as 20% of the cases were defined as postoperative PE. Thromboembolism is recognized as the leading cause of preventable in-hospital deaths [14]. At our institution, a protocol that mandates risk stratification and prophylaxis for thromboembolism was set in place. However, there remains insufficient data on the rate of adherence to this protocol. A previous study involving seven major hospitals in Saudi Arabia found a gap between guidelines and practice of thromboprophylaxis measures [15]. It has been conclusively shown that missing enoxaparin doses during hospitalization increases the incidence of deep venous thrombosis [16].

Notably, 9% of patients with PE in this study had sickle cell disease. While the prevalence of sickle cell disease varies significantly across Saudi Arabia, it is reported to be the highest in the Eastern Province (17%), where the current study was based [2]. Such a high prevalence might be explained, at least partly, by the high level of consanguineous marriages in this population [17]. This study highlights two features related to PE in patients with sickle cell disease. First, these patients tend to have PE at a younger age, which may increase the morbidity of the disease, as these patients might require lifelong treatment. Second, PE in these patients tends to occur in the peripheral vasculature. In their analysis of radiographic studies in patients with sickle cell disease, Gopalsamy et al. [18] similarly showed that the segmental vessels were involved in 75% of PE cases. This finding is not particularly surprising given that sickle cell disease predominantly affects the microcirculation and small vessels. However, a considerable volume of recent literature suggests that hypercoagulability plays a crucial role in thrombotic events in sickle cell disease [19]. It is also important to note that acute chest syndrome, a common complication of sickle cell disease [20], can be clinically indistinguishable from PE. Patients with sickle cell disease thus more frequently undergo CT-PA scans, which might diagnose clinically insignificant incidental PE.

We also saw several cases with a previous history of venous thromboembolism, which were in a younger age group than the other cases. This might be related to the presence of undiagnosed inherited thrombophilia. Testing for thrombophilia in our study, however, was limited, given that it does not significantly alter the management or outcomes in most cases, and is not one of the recommended routine tests [21]. Smoking was also a risk factor in this study, predominantly in men. However, smoking may have been underreported in women since there is social stigma around women smokers in the community.

As in the PIOPED study [22], dyspnea was the most common presentation among our patients. Chest pain in our study (36.5%), however, seemed to be less common than in the PIOPED study (66%) [22]. Consistent with recent studies, hemoptysis was an uncommon presentation. Older studies reported hemoptysis rates as high as 28% [23], likely because of delayed diagnosis before CT-PA scans became widely available.

This study did not find any difference in the clinical presentation between central and peripheral PE. The exception to this was hemoptysis, which was significantly more common in patients with isolated subsegmental PE. Garcia-Sanz et al. [24] suggested that hemoptysis was more common in subsegmental PE than in central PE, although the difference was not statistically significant. Hemoptysis is known to occur in peripheral PE because of alveolar hemorrhage that results from the influx of high-pressure blood from bronchial circulation of the obstructed area [25].

### Imaging profile

#### False-positive pulmonary embolism

Out of the 103 non-negative CT-PA scans, 12 scans were negative for PE. All these patients were assumed to have peripheral PE. Although the radiology reports were not definitive in all cases, anticoagulant therapy was administered in two-thirds of the patients.

Inconsistency in the interpretation of the CT-PA scans has been well described before. For example, a retrospective review, performed over 12 months at a tertiary-care university hospital, found a discordance rate of 25% between the original interpretation and retrospective review [26]. We are currently in the process of analyzing this interpretive discordance in the diagnosis of PE with different vascular involvement between radiologists with varying training levels.

### Chest radiography

Chest radiography was performed in almost all patients with PE. Most patients had non-specific changes, such as parenchymal infiltrates. However, the main role of chest radiography in patients with suspected PE is to look for alternative diagnoses that explain the patient’s symptoms, rather than to establish a diagnosis of PE.

### Pleuroparenchymal CT-PA findings

More than 25% of the PE cases were associated with pleural effusion. The development of pleural effusion in PE remains unclear, but could be associated with increased permeability of the pulmonary capillaries [27]. The frequency of pleural effusion found in the CT-PA scans in this study was consistent with the frequency seen in previous studies [28, 29]. Moreover, the impacts of pleural effusion on outcomes in patients with PE showed conflicting results [28, 30].

### Study limitations

This study has certain limitations. It was conducted in a single-academic institution, yielding a small sample size that was not adequate for multivariable regression analysis. The retrospective observational nature of the study is another limitation. Furthermore, the study did not include CT-PA scans that were performed for purposes other than ruling out PE. Such scans could have identified incidental PE.

## Conclusion

This study provides insights into the clinical and radiological profiles of patients with PE in Saudi Arabia. It suggests that clinical and imaging profiles in patients with peripheral PE are similar to those in patients with central PE. Patients with sickle cell disease and obesity constituted a significant proportion of all PE cases in this study. Future research should focus on the clinical value of peripheral PE in patients with sickle cell disease.

## Data Availability

The datasets used and/or analyzed during the current study are available from the corresponding author on reasonable request.

## Abbreviations

CI: Confidence interval
CCI: Charlson Comorbidity Index
CT-PA: Computed tomography pulmonary angiography
ED: Emergency department
IQR: Interquartile range
OR: Odds ratio
PE: Pulmonary embolism
PESI: Pulmonary Embolism Severity Index
PIOPED: Prospective Investigation of Pulmonary Embolism

## References

[1] Horlander KT, Mannino DM, Leeper KV (2003). Pulmonary embolism mortality in the United States, 1979-1998: an analysis using multiple-cause mortality data. Arch Intern Med.

[2] Jastaniah W (2011). Epidemiology of sickle cell disease in Saudi Arabia. Ann Saudi Med.

[3] Naik RP, Streiff MB, Lanzkron S (2013). Sickle cell disease and venous thromboembolism: what the anticoagulation expert needs to know. J Thromb Thrombolysis.

[4] Beenen LFM, Bossuyt PMM, Stoker J, Middeldorp S (2018). Prognostic value of cardiovascular parameters in computed tomography pulmonary angiography in patients with acute pulmonary embolism. Eur Respir J.

[5] Karabulut N, Kiroglu Y (2008). Relationship of parenchymal and pleural abnormalities with acute pulmonary embolism: CT findings in patients with and without embolism. Diagn Interv Radiol.

[6] Meinel FG, Nance JW, Schoepf UJ, Hoffmann VS, Thierfelder KM, Costello P (2015). Predictive value of computed tomography in acute pulmonary embolism: systematic review and meta-analysis. Am J Med.

[7] Charlson ME, Pompei P, Ales KL, MacKenzie CR (1987). A new method of classifying prognostic comorbidity in longitudinal studies: development and validation. J Chronic Dis.

[8] Aujesky D, Obrosky DS, Stone RA, Auble TE, Perrier A, Cornuz J (2005). Derivation and validation of a prognostic model for pulmonary embolism. Am J Respir Crit Care Med.

[9] Elias A, Mallett S, Daoud-Elias M, Poggi JN, Clarke M (2016). Prognostic models in acute pulmonary embolism: a systematic review and meta-analysis. BMJ Open.

[10] Smith SB, Geske JB, Kathuria P, Cuttica M, Schimmel DR, Courtney DM (2016). Analysis of national trends in admissions for pulmonary embolism. Chest.

[11] Naess IA, Christiansen SC, Romundstad P, Cannegieter SC, Rosendaal FR, Hammerstrom J (2007). Incidence and mortality of venous thrombosis: a population-based study. J Thromb Haemost.

[12] Al-Ghamdi S, Shubair MM, Aldiab A, Al-Zahrani JM, Aldossari KK, Househ M (2018). Prevalence of overweight and obesity based on the body mass index; a cross-sectional study in Alkharj, Saudi Arabia. Lipids Health Dis.

[13] Christiansen SC, Lijfering WM, Naess IA, Hammerstrom J, van Hylckama VA, Rosendaal FR (2012). The relationship between body mass index, activated protein C resistance and risk of venous thrombosis. J Thromb Haemost.

[14] Geerts WH, Bergqvist D, Pineo GF, Heit JA, Samama CM, Lassen MR (2008). Prevention of venous thromboembolism: American College of Chest Physicians Evidence-Based Clinical Practice Guidelines (8th edition). Chest.

[15] Al-Hameed FM, Al-Dorzi HM, Qadhi AI, Shaker A, Al-Gahtani FH, Al-Jassir FF (2017). Thromboprophylaxis and mortality among patients who developed venous thromboembolism in seven major hospitals in Saudi Arabia. Ann Thorac Med.

[16] Louis SG, Sato M, Geraci T, Anderson R, Cho SD, Van PY (2014). Correlation of missed doses of enoxaparin with increased incidence of deep vein thrombosis in trauma and general surgery patients. JAMA Surg.

[17] Warsy AS, Al-Jaser MH, Albdass A, Al-Daihan S, Alanazi M (2014). Is consanguinity prevalence decreasing in Saudis?: a study in two generations. Afr Health Sci.

[18] Gopalsamy SN, El Rassi F, McLemore ML (2017). Pulmonary embolism in sickle cell disease: retrospective analysis of the use and yield of radiographic studies. Blood.

[19] Shet AS, Wun T (2018). How I diagnose and treat venous thromboembolism in sickle cell disease. Blood.

[20] Pahl K, Mullen CA (2016). Original research: acute chest syndrome in sickle cell disease: effect of genotype and asthma. Exp Biol Med (Maywood).

[21] Stern RM, Al-Samkari H, Connors JM (2019). Thrombophilia evaluation in pulmonary embolism. Curr Opin Cardiol.

[22] Stein PD, Terrin ML, Hales CA, Palevsky HI, Saltzman HA, Thompson BT (1991). Clinical, laboratory, roentgenographic, and electrocardiographic findings in patients with acute pulmonary embolism and no pre-existing cardiac or pulmonary disease. Chest.

[23] Dalen JE, Haffajee CI, Alpert JS, Howe JP, Ockene IS, Paraskos JA (1977). Pulmonary embolism, pulmonary hemorrhage and pulmonary infarction. N Engl J Med.

[24] Garcia-Sanz MT, Pena-Alvarez C, Lopez-Landeiro P, Bermo-Dominguez A, Fonturbel T, Gonzalez-Barcala FJ (2014). Symptoms, location and prognosis of pulmonary embolism. Rev Port Pneumol.

[25] Righini M, Robert-Ebadi H, Le Gal G (2017). Diagnosis of acute pulmonary embolism. J Thromb Haemost.

[26] Hutchinson BD, Navin P, Marom EM, Truong MT, Bruzzi JF (2015). Overdiagnosis of pulmonary embolism by pulmonary CT angiography. AJR Am J Roentgenol.

[27] Light RW: Pleural effusion in pulmonary embolism. In: *Semin Respir Crit Care Med: 2010*: © Thieme Medical Publishers; 2010: 716-722. 10.1055/s-0030-1269832 21213203

[28] Kiris T, Yazici S, Koc A, Koprulu C, Ilke Akyildiz Z, Karaca M (2017). Prognostic impact of pleural effusion in acute pulmonary embolism. Acta Radiol.

[29] Zhou X, Zhang Z, Zhai Z, Zhang Y, Miao R, Yang Y (2016). Pleural effusions as a predictive parameter for poor prognosis for patients with acute pulmonary thromboembolism. J Thromb Thrombolysis.

[30] Choi SH, Cha SI, Shin KM, Lim JK, Yoo SS, Lee SY (2017). Clinical relevance of pleural effusion in patients with pulmonary embolism. Respiration.

